# Sex-Specific Mortality from Asbestos-Related Diseases, Lung and Ovarian Cancer in Municipalities with High Asbestos Consumption, Brazil, 2000–2017

**DOI:** 10.3390/ijerph19063656

**Published:** 2022-03-19

**Authors:** Cézar Akiyoshi Saito, Marco Antonio Bussacos, Leonardo Salvi, Carolina Mensi, Dario Consonni, Fernando Timoteo Fernandes, Felipe Campos, Franciana Cavalcante, Eduardo Algranti

**Affiliations:** 1 Fundacentro, Ministério do Trabalho e Previdência, São Paulo 05409-002, Brazil; cezar.saito@fundacentro.gov.br (C.A.S.); bussacos@fundacentro.gov.br (M.A.B.); fernando.fernandes@fundacentro.gov.br (F.T.F.); 2 Programa de Saúde Ambiental e de Saúde do Trabalhador, Instituto de Saúde Coletiva, Universidade Federal da Bahia, Salvador 40110-040, Brazil; leomaxil@hotmail.com (L.S.); fcampos1205@gmail.com (F.C.); francicavalcante@hotmail.com (F.C.); 3 Occupational Health Unit, Fondazione IRCCS Ca’ Granda Ospedale Maggiore Policlinico, 20122 Milan, Italy; dario.consonni@unimi.it

**Keywords:** asbestos, Brazil, mortality

## Abstract

The aim of this study is to compare the mortality rates for typical asbestos-related diseases (ARD-T: mesothelioma, asbestosis, and pleural plaques) and for lung and ovarian cancer in Brazilian municipalities where asbestos mines and asbestos-cement plants had been operating (areas with high asbestos consumption, H-ASB) compared with in other municipalities. The death records for adults aged 30+ years were retrieved from multiple health information systems. In the 2000–2017 time period, age-standardized mortality rates (standard: Brazil 2010) and standardized rate ratios (SRR; H-ASB vs. others) were estimated. The SRRs for ARD-T were 2.56 for men (257 deaths in H-ASB municipalities) and 1.19 for women (136 deaths). For lung cancer, the SRRs were 1.33 for men (32,604 deaths) and 1.19 for women (20,735 deaths). The SRR for ovarian cancer was 1.34 (8446 deaths). Except for ARD-T and lung cancer in women, the SRRs were higher in municipalities that began using asbestos before 1970 than in municipalities that began utilizing asbestos from 1970 onwards. In conclusion, the mortality rates for ARD-T, and lung and ovarian cancer in municipalities with a history of asbestos mining and asbestos-cement production exceed those of the whole country. Caution is needed when interpreting the results of this ecological study. Analytical studies are necessary to document the impact of asbestos exposure on health, particularly in the future given the long latency of asbestos-related cancers.

## 1. Introduction

Mesothelioma, asbestosis, and pleural plaques are considered typical asbestos-related diseases (ARD-T). Although other elongated mineral particles—erionite, winchite, and fluoro-edenite—are also causally related to mesothelioma [1,2,3], their geological occurrences are limited, and they do not have commercial applications. Mesothelioma is considered the fingerprint of asbestos usage.

Between 1961 and 2017, more than 8 million tons of chrysotile and small amounts of anthophyllite were produced and about 7 million tons of asbestos was consumed in Brazil [4], including amphibole imports, which took place up to the 1980s [5]. However, chrysotile had the highest usage. Overall, the internal consumption of asbestos reached a peak from the mid-1980s to the 1990s, following which usage declined until its ban in 2017. Asbestos-cement products accounted for more than 98% of the national consumption [6].

To date, in Brazil, the sum of deaths due to ARD-T amount to less than 100 cases per year on average. Specifically, mesothelioma mortality rates are close to background rates in areas where there is no apparent fiber consumption [7].

Mesothelioma has an attributable fraction (AF) to asbestos at around 90% in occupationally exposed men, a figure which is lower in women. However, the AF related to non-occupational exposures is higher in women [8]. The lower AF in women is partly due to an inadequate capture of exposure histories caused by lack of knowledge about previous exposure rather than due to biological differences [9,10]. In fact, most studies on the effect of asbestos have been performed in sectors with a predominance of male workers; it was only in recent years that patterns of asbestos exposure have been demonstrated in other industries (e.g., the textile industry, in which women were the majority employed and affected) [10].

Asbestosis usually occurs in individuals with high cumulative exposures; therefore, it is almost always associated with occupational exposure [11]. Pleural plaques are the most frequent disease associated with asbestos [12] but are rarely an underlying cause of death [13]. However, they also have other causes, such as the evolution of pleural empyema, pleural tuberculosis, and chest trauma, which can be easily discerned through the patient’s clinical history.

In addition to mesothelioma, asbestos is also recognized as a causal agent of lung and ovarian cancer [14,15]. In 2020, lung cancer was the most lethal malignant neoplasm in men and the second most lethal in women in Brazil [16]. Asbestos-related AF for cancers other than mesothelioma vary according to the type of industrialization in the country or the characteristics of the area being examined [17,18]. In occupationally exposed populations, asbestos-related lung cancer is more common than mesothelioma. It is estimated that the mesothelioma-to-lung-cancer ratio varies from 1:1 to 1:10, depending on fiber type and type of industry. In terms of chrysotile, which is widely used in Brazil, it is likely that the number of lung cancer cases largely exceeds the number of mesothelioma cases [19]. An ecological analysis of lung cancer deaths in the municipality of Osasco--where the largest asbestos-cement plant in Latin America operated—has shown that the trend in lung cancer mortality in men aged 60 years and over continued to rise during the time period considered in the study, in contrast to a fall in the state of São Paulo [20]. As for ovarian cancer, it had an incidence of 6650 cases and 4123 deaths in 2020 in Brazil [16]. The differential diagnosis between ovarian cancer and peritoneal mesothelioma can be challenging, even with the recent advent of immunohistochemical techniques [21]. However, peritoneal mesotheliomas are quite rare.

Between 2015 and 2020, we conducted a national project called Interdisciplinary Project of Occupational Asbestos Exposure and its Health Effects. One of the objectives was to capture all the asbestos-related death records in Health Information System (HISs), linking databases and constituting a single database. The linking process proved to be adequate [22,23] and allowed us to estimate that the number of ARD-T deaths in the Brazilian Mortality Information System increased by an average of 33% [24].

The objective of this work is to compare the mortality rates for ARD-T—pooled and as individual diagnosis, mesothelioma, asbestosis, and pleural plaques—as well as lung and ovarian cancer in a group of municipalities where there was high asbestos consumption (H-ASB) when compared with rates from other Brazilian municipalities.

## 2. Materials and Methods

This is a retrospective study of deaths from ARD-T, and lung and ovarian cancer recovered from at least one HIS in Brazil, between 2000 and 2017, in individuals aged 30 years or over. Five municipalities that had asbestos mining and twenty-four municipalities that housed asbestos-cement plants were considered high consumption areas (Supplementary Table S1) and were compared to the remaining Brazilian municipalities as a reference

### 2.1. Data Sources

A total of five HISs and a repository had the required study data: (a) the Brazilian mortality information system, (b) public and private hospital admission databases, (c) hospital cancer registries from the National Cancer Institute (INCA), and (d) the compulsory disease notification system. All HISs are anonymous and publicly available. In addition, a repository of ARD-T and other ARD cases from outpatient units that specialized in occupational respiratory diseases was used, comprising Fundacentro, Instituto do Coração/USP and the Center for the Study of Occupational Health and Human Ecology (CESTEH/FIOCRUZ). Detailed information can be found elsewhere [24].

Disease records are coded in each HIS according to ICD-10, including specific codes for malignant mesothelioma (C45X) in the pleura (C45.0), the peritoneum (C45.1), the pericardium (C45.2), and other (C45.7) and unspecified (C45.9) locations; for asbestosis (J61); for asbestos-related pleural plaques (J92.0); for lung cancer (C34X); and for ovarian cancer (C56X). The availability of information in the data sources covered different periods. SIM and SIH-SUS had data available for the entire study period, CIH-CIHA had data available for the period between 2008 and 2017, INCA had data available for the period between 2000 and 2014, SINAN had data available for the period between 2006 and 2017, and outpatient units had data available for the period from 2000 to 2016.

The INCA uses the International Classification of Diseases for Oncology (ICD-O), in which diagnoses are defined by the topography and morphology of the neoplasm. In ICD-O-3, mesothelioma morphology corresponded to codes 9050/3 (malignant mesothelioma not otherwise specified), 9051/3 (malignant fibrous mesothelioma), 9052/3 (malignant epithelioid mesothelioma), and 9053/3 (malignant biphasic mesothelioma). Deaths with the abovementioned codes were analyzed together with those of the C45X group of the ICD-10 and classified according to topographic location.

### 2.2. ARD-T, Lung and Ovarian Cancer Databases

The strategy for creating the databases was described elsewhere for mesothelioma/pleural cancer and for laryngeal cancer [22,23]. Briefly, in each HIS database, death records of interest were selected based on ICD-10 codes. The completeness and consistency of the records were reviewed, the variable formats were standardized, and corrections and imputations were made if the requisite data were available. For each individual record, the data source (HIS) was registered. A common key variable for linkage was defined, using sex, date of birth, year of death, and the code of the city of residence, to create an algorithm that allowed for the anonymous pairing of records from the same individual. Records with missing identification variables were excluded.

Except for the hospital cancer registry and the disease notification system, the other databases reported the underlying cause of death or the main diagnosis, and the contributing causes or comorbidity that could result in more than one registered diagnosis of interest to a single death. In this case, the decision criteria adopted was, first, to assume the underlying cause or the main diagnosis; second, to assume the contributory or comorbidity cause in the absence of an underlying cause of interest; third, when there was more than one diagnosis of interest, irrespective of being a contributory or secondary cause, with the oncological diagnosis prevailing over the non-malignant diseases; and fourth, data from the repository of specialized outpatient clinics had clinical imaging and, when available, anatomopathological information, when different from the HIS databases, was chosen for analysis.

For lung and ovarian cancer, records were extracted from the same databases except the repository, using the same approach. However, the compulsory disease notification system did not include any of the two cancers, as they are not subject to compulsory notification unless they are considered occupationally related.

Population data include the Brazilian 2000 and 2010 census data and intercensal population estimates [25].

### 2.3. Statistical Analysis

For each disease or group of diseases (ARD-T), we calculated yearly (2000–2017) and overall crude and age-standardized rates (ASRs, per million person-years) for the 29 municipalities (H-ASB) and the reference group. The standard population was the Brazilian 2010 population ≥ 30 years of age in 10-year age categories.

Standardized rate ratios (SRR) and 95% confidence intervals (95% CI) of H-ASB against the rest of the country as well as of H-ASB municipalities that started consumption before 1970 against those that started from 1970 onwards were calculated. Statistical analysis was performed with Stata Statistical Software: Release v17 (StataCorp. LLC, College Station, TX, USA, 2021) using the command “distrate” [26].

## 3. Results

The population aged 30 years or over in the 29 H-ASB municipalities ranged from 2265 inhabitants in Jaramataia to 3,620,301 in the city of Rio de Janeiro. Combined, the 29 municipalities totaled 9,725,764 inhabitants, representing 10.4% of the Brazilian population according to the 2010 census.

**Figure 1:**
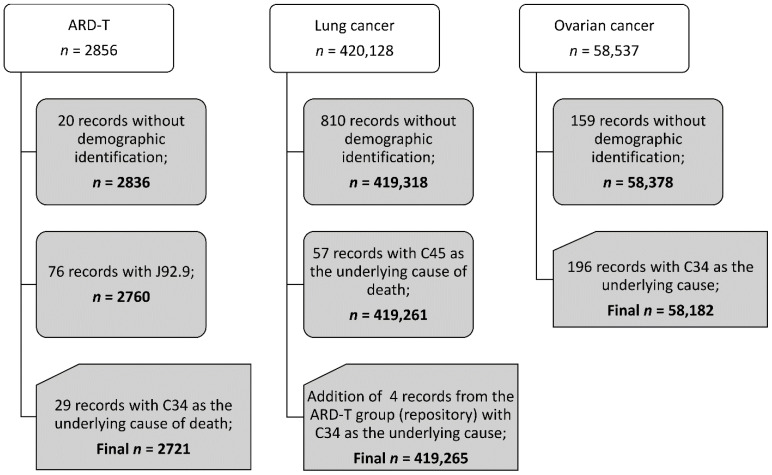
Records for analysis in adults of 30 years of age and older, Brazil, 2000–2017.

We found that there were 2721 pooled ARD-T, 419,265 lung cancer, and 58,182 ovarian cancer death records between 2000 and 2017. Figure 1 presents the study death data structure according to the diagnosis. Supplementary Table S1 displays the number of pooled ARD-T, and lung and ovarian cancer deaths registered for each of the 29 H-ASB (and for all other Brazilian municipalities (*n* = 5539).

In the H-ASB, 493 deaths from ARD-T were retrieved (357 in men and 136 in women), corresponding to 18.1% of ARD-T death records (Table 1). From these, 308 (62.5%) had mesothelioma records. Of deaths with mesothelioma records, pleural mesothelioma comprised 47% and 37.7% in men and women, whereas peritoneal mesothelioma comprised 9.8% and 25.4%, respectively. The proportions for Brazil were similar. Among the ARD-T cases in the H-ASB, the men-to-women ratios were 1.7 for mesothelioma and 7.4 for non-malignant ARD-T. The ratios were 1.4 and 2.0 for the remaining municipalities, respectively.

**Figure 2:**
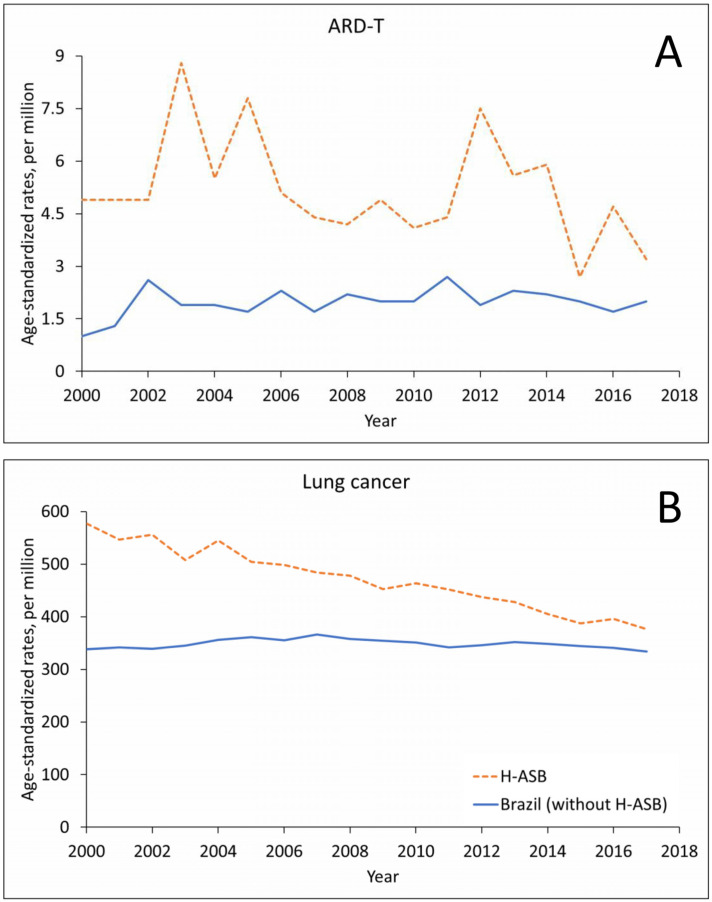
Age-standardized rates (ASRs, per million; standard population: Brazil 2010) in the 29 H-ASB municipalities (dashed lines) and in all other municipalities (solid lines) by year of death for men 30 years and over in Brazil, 2000–2017. (**A**) Pooled ARD-T. (**B**) Lung cancer.

The number of ARD-T death records in men remained stable in the H-ASB and increased in Brazil in the 18-year period, with oscillating trends in crude- and age-standardized rates (Figure 2A and Supplementary Table S2). Lung cancer showed an increase in numbers for both the H-ASB and Brazil but with decreasing rates in the H-ASB and stable rates in Brazil (Figure 2B and Supplementary Table S2).

**Figure 3:**
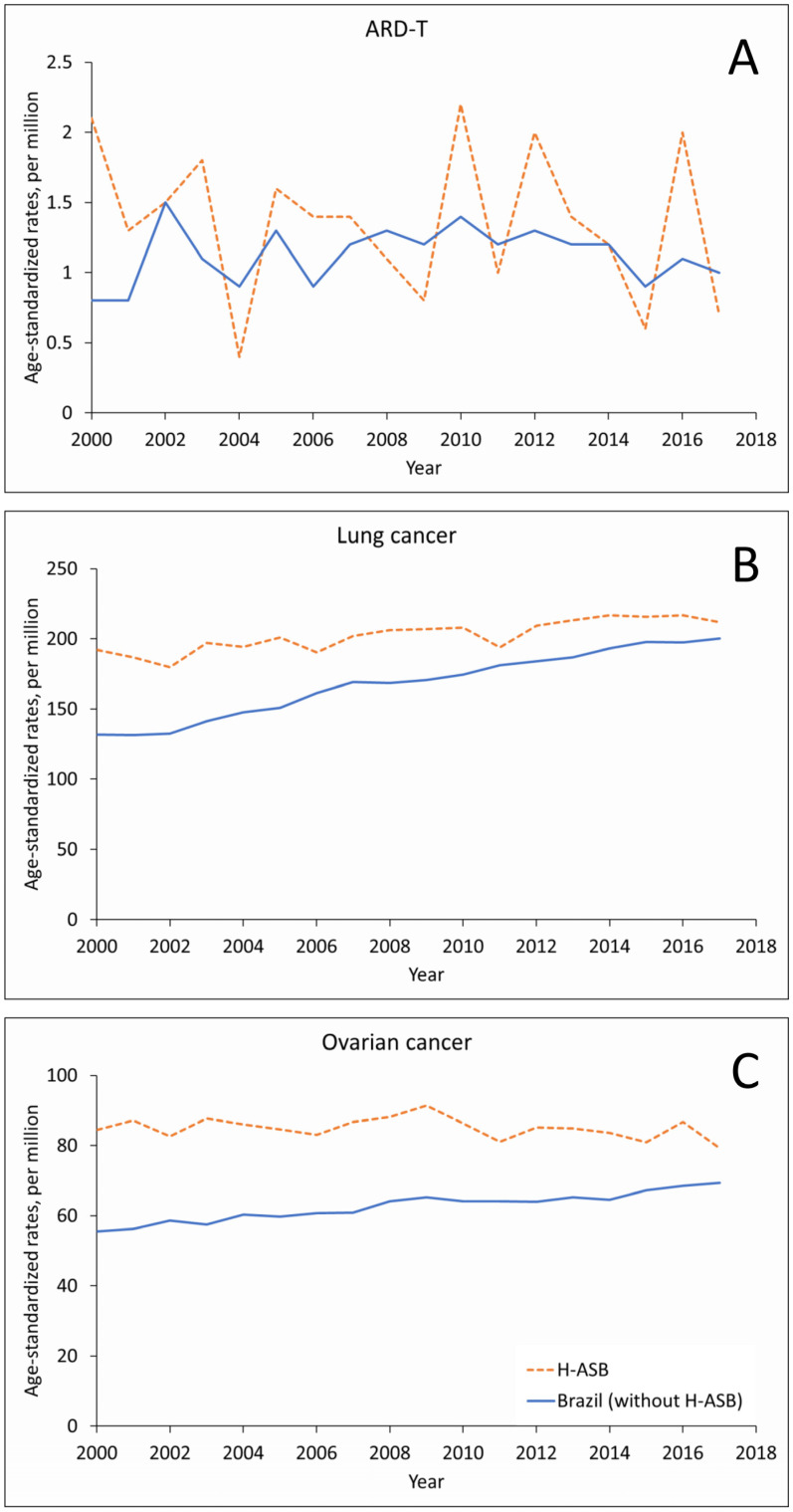
Age-standardized rates (ASRs, per million; standard population: Brazil 2010) in the 29 H-ASB (dashed lines) and in the reference group (all other municipalities, solid lines) by year of death for women 30 years and over in Brazil, 2000–2017. (**A**) Pooled ARD-T. (**B**) Lung cancer. (**C**) Ovarian cancer.

In women, the small number of ARD-T deaths fluctuated in the 29 H-ASB municipalities and increased in the reference group, with increasing trends for the number of lung cancer deaths and rates (Figure 3A,B and Supplementary Table S3). Ovarian cancer showed increasing trends in numbers, with stable rates in the H-ASB and increasing rates in the reference municipalities (Figure 3C, Supplementary Table S3).

During the whole observation period, ASRs for all outcomes were systematically higher in the H-ASB municipalities in both genders (Table 2). In particular, in men, SRR ranged from 1.70 to 6.35 for ARD-T and an SRR of 1.33 was observed for lung cancer. For women, all SRRs for ARD-T (except pleural plaques) and for lung cancer were elevated (range: 1.17–1.19). Ovarian cancer showed a 34% excess.

Municipalities that started consumption before 1970 showed a different gender behavior for ARD-T when compared to those that started from 1970 onwards (Table 3). In men, the SRR was 3.26 for pre-1970 and 1.30 for 1970 onward, while in women, the corresponding figures were 1.10 and 1.35, respectively. For lung cancer, in men, there were 40% and 19% excess deaths for pre-1970 and 1970 onwards, respectively, but in women, the excess was the same in both periods (19%). For ovarian cancer, SRR was slightly higher before 1970.

## 4. Discussion

In Brazil, in the period 2000–2017, age-standardized mortality rates of pooled ARD-T, and lung and ovarian cancer were higher in 29 municipalities with high asbestos use compared with the rest of the country.

A recent analysis of the ARD-T database spanning from 2008 to 2014 showed that the capture of records from hospital admissions, hospital cancer registries, compulsory disease registry, and the repository added 1/3 of deaths with mesothelioma records and almost 40% with asbestosis and pleural plaques records to those registered in the mortality information system, which was the main repository of records. The capture of underreported cases showed no clear trend, possibly because of the short period of evaluation [24]. In the present study, the same strategy was used to capture ARD-T records.

Global temporal trends in incidence and mortality from mesothelioma and asbestosis between 1990 and 2017 have shown increasing incidence of and mortality from mesothelioma in low sociodemographic index (SDI) countries, especially among females [27], and an increasing incidence rate of asbestosis that is more pronounced in developed countries [28]. It is known that asbestos health effects are not limited to occupational settings [29,30]. In the high consumption areas, the bulk of occupational exposure in mining and asbestos-cement plants in which male workers predominate was reflected by the 7.4 men/women ratio of asbestosis and pleural plaques records. In contrast, the 1.7 men/women ratio for mesothelioma was lower.

Mesothelioma incidence in women correlates with areas of high asbestos consumption, possibly because of environmental exposure [31], which could be due to dust emissions from nearby plants, dumping or waste sites, or domestic asbestos from water containers and/or roof tiles from asbestos-cement products. Ferrante et al. showed a two-fold risk of mesothelioma for living in the vicinity of environmental sources and for domestic exposure in male and female residents of Casale-Monferrato, Italy. The risk of mesothelioma increased as non-occupational cumulative exposure increased, and what was worrying was the increased risk at cumulative exposure estimates as low as 1 f/mL-year [32]. In the Italian mesothelioma national surveillance system, malignant mesothelioma due to non-occupational exposure represented 10% of overall cases. Of these, environmental and domestic exposures tended to be more prevalent in women [33] although at lower levels compared to those reported in workplaces. Similar findings were described in the municipality of Broni, Italy: of 147 MM cases registered between 2000 and 2011, only 38 were occupationally related, and of the non-occupational cases, domestic asbestos exposure prevailed in women while male exposure was primarily related to the environmental [34].

In the city of Amagasaki, Japan, where a cement pipe factory has been operating since 1957, a cause-specific mortality study among residents showed an elevated Standardized Mortality Ratio for mesothelioma and lung cancer in men and women. These figures were relative to the matched country population but with a substantial sex difference [35]. Another population-based nested case–control study in Amagasaki, adjusted for occupational exposure, demonstrated an increased and similar risk for mesothelioma in both sexes according to quintiles of neighborhood exposure [36]. Environmental exposure to asbestos in Danish former school children that studied in four schools nearby an asbestos-cement plant, both with or without further domestic or occupational exposures, showed a strong association with the incidence of malignant mesothelioma [37].

Recent studies have shown geographic correlation of incidence of mesothelioma and ovarian cancer in the U.S. [38] and in Lombardy, Italy [39]. In the U.S., the correlation remained similar when incidental cases of mesothelioma were restricted to pleural mesothelioma, which constituted in an argument favoring the causal relationship between exposure to asbestos and ovarian cancer, since it overcomes bias raised from potential misclassification of diagnosis of peritoneal mesothelioma and ovarian cancer, commonly difficult to distinguish at late stages. Among the types of ovarian cancer, only the histological types of ovarian stromal tumors and germ cell tumors did not correlate with the incidence of mesothelioma [38]. In Lombardy, by using Bayesian hierarchical shared models, it was possible to demonstrate a common risk factor for cancer of the pleura and ovary at small geographical units [39].

Chrysotile was the predominant fiber type used in Brazil. Despite being associated with a lower risk of mesothelioma, it is associated with lung, laryngeal, and ovarian cancer [40]. Moreover, there is no convincing evidence that the risk of lung cancer varies with fiber type [41]. The recent asbestos ban in Brazil reduced occupational exposure but it will not reduce future risks related to living with and/or manipulating in-place asbestos or being in the vicinity of asbestos dumpings, as in communities where there was high fiber consumption [42].

This study must be evaluated within its limitations. First, this is an ecological study in which exposure assignment was based only on residence in municipalities with high asbestos use and which brought together small municipalities (e.g., Jaramataia) and large cities (e.g., Rio de Janeiro). Therefore, the analysis of lung and ovarian cancer could not take into account the role of potential confounders. Ecological analyses using aggregated data can lead to statistically larger or even to spurious correlations when compared with individual data [43].

It is likely that ARD-T cases, mainly malignancies, have sought better diagnosis and treatment and migrated from smaller municipalities to cities or metropolitan areas where high complexity or specialized health care are located. The 18.1% of deaths from ARD-T in the H-ASB municipalities, which represented 10.4% of the country’s population, increased to over 45% when including the metropolitan neighboring areas (results not shown).

Another limitation refers to the timeline of asbestos consumption in the H-ASB municipalities. For example, the asbestos-cement industry started activities in the 1940s in the municipalities of Osasco and São Caetano do Sul and only in the 1970s in Pedro Leopoldo, Capivari, Colombo, and Londrina. Possibly, this timing was reflected both in changes in occupational exposure levels over the years and in the magnitude of non-occupational exposures of the resident populations. Consequently, the period under review, 2000–2017, reflects different occupational and environmental exposure conditions in each of the municipalities. Fourteen out of the twenty-nine municipalities started asbestos consumption before 1970 and were responsible for 75.9% of deaths with ARD-T records and 2/3 of deaths of lung and ovarian cancer. When stratifying for municipalities that started consumption before or from 1970 onwards, different patterns were observed in the two genders. Except for ARD-T and lung cancer in women, SRRs were higher in municipalities that started asbestos use before 1970 than in municipalities that started later on. Apart from the repository, where clinical data was available, diagnoses were based in the HIS records only and their accuracy could not be checked.

Finally, the choice of cities that housed mining and asbestos-cement plants--although responsible for all asbestos production and more than 98% of fiber consumption--may not reflect the frequency and magnitude of occupational exposure in other sectors or industries that made use of asbestos or had it installed in the premises.

## 5. Conclusions

Despite capturing death records from several HISs, mortality rates for a composite of mesothelioma, asbestosis, and pleural plaques remained low. However, even with the study limitations associated with the underdiagnosis of ARD-T in areas of long-term history of high asbestos consumption, we found excess mortality from ARD-T, which is paralleled with an excess of lung cancer deaths in both sexes and ovarian cancer deaths in women. Caution is needed in interpreting the results of this ecological study. Analytical studies are necessary to document the health impact of asbestos use, also in future years, given the long latency of asbestos-related cancers and given that asbestos use has continued until recently in many municipalities.

## Figures and Tables

**Table 1:** Number of deaths (*n*) with records of ARD-T, and lung and ovarian cancer in the 29 municipalities (H-ASB) and for all Brazilian municipalities, 2000–2017.

Disease (ICD-10 Code)	Men		Women	
	H-ASB	Brazil	H-ASB	Brazil
	*n*	*n*	*n*	*n*
Typical asbestos-related diseases (ARD-T)	357	1686	136	1035
Mesothelioma (C45X)	194	1253	114	881
Mesothelioma of pleura (C45.0)	91	563	43	318
Mesothelioma of peritoneum (C45.1)	19	173	29	212
Mesothelioma of pericardium (C45.2)	3	29	1	13
Mesothelioma of other sites (C45.7)	16	75	5	59
Mesothelioma, unspecified (C45.9)	65	413	36	279
Asbestosis (J61)	97	244	14	73
Pleural plaques (J92.0)	66	189	8	81
Lung cancer (C34)	32,604	261,816	20,735	157,449
Ovarian cancer (C56)	-	-	8446	58,182

**Table 2:** Number of deaths (*n*), from pooled and individual ARD-T, and lung and ovarian cancer in the H-ASB municipalities and the standardized rate ratios (SRR) and 95% confidence intervals (CI) compared to the reference group, Brazil, 2000–2017. Standard population: Brazil 2010.

Disease (ICD-10 Code)	Men			Women		
	*n*	SRR	95% CI	*n*	SRR	95% CI
Typical asbestos-related diseases (ARD-T)	357	2.56	2.27–2.88	136	1.19	0.99–1.43
Mesothelioma (C45X)	194	1.70	1.45–1.99	114	1.17	0.95–1.43
Asbestosis (J61)	97	6.35	4.86–8.28	14	1.86	0.96–3.39
Pleural plaques (J92.0)	66	5.06	3.69–6.89	8	0.86	0.35–1.79
Lung cancer (C34)	32,604	1.33	1.31–1.34	20,735	1.19	1.17–1.20
Ovarian cancer (C56)	-	-	-	8446	1.34	1.31–1.37

Abbreviations: SRR, standardized rate ratios; CI, confidence interval.

**Table 3:** Number of deaths (*n*), standardized rate ratios (SRR), and 95% confidence intervals (CI) of pooled ARD-T, and lung (by sex) and ovarian cancer (in women) in the H-ASB municipalities that started consumption before (<70) or after (70+) 1970 in Brazil, 2000–2017. Standard population: Brazil 2010.

	**Men**					
	**<1970**			**1970+**		
	***n***	**SRR**	**95% CI**	***n***	**SRR**	**95% CI**
Typical asbestos-related diseases (ARD-T)	291	3.26	2.86–3.70	66	1.30	0.99–1.67
Lung cancer	22,021	1.40	1.38–1.42	10,583	1.19	1.17–1.22
	**Women**					
	**<1970**			**1970+**		
	***n***	**SRR**	**95% CI**	***n***	**SRR**	**95% CI**
Typical asbestos-related diseases (ARD-T)	83	1.10	0.87–1.38	53	1.35	1.01–1.79
Lung cancer	13,697	1.19	1.17–1.21	7038	1.19	1.17–1.22
Ovarian cancer	5699	1.39	1.35–1.42	2747	1.26	1.21–1.31

Abbreviations: SRR, standardized rate ratios; CI, confidence interval.

## Data Availability

Patient consent was waived due to the use of anonymized public data. Data from the HISs used in this study are publicly available at DATASUS (https://datasus.saude.gov.br/transferencia-de-arquivos/) (accessed on 27 July 2021) and INCA^16^. Data from the outpatient repository can be made available only after request to the authors and after anonymization.

## Supplementary Materials

The following supporting information can be downloaded at https://www.mdpi.com/1660-4601/19/6/3656/s1, Table S1: Population (2010) and number of deaths with records of ARD-T, and lung and ovarian cancer in the H-ASB municipalities (and respective Brazilian State), Brazil, 2000–2017; Table S2: Crude and age-standardized rates (ASRs, per million) in the 29 municipalities (H-ASB) and in Brazil (without H-ASB) by year of death in men 30 years and over in Brazil, 2000–2017; Table S3: Crude and age-standardized rates (ASRs, per million) in the 29 municipalities (H-ASB) and in Brazil (without H-ASB) by year of death in women 30 years and over in Brazil, 2000–2017.
