# Correction: Saito et al. Sex-Specific Mortality from Asbestos-Related Diseases, Lung and Ovarian Cancer in Municipalities with High Asbestos Consumption, Brazil, 2000–2017. *Int. J. Environ. Res. Public Health* 2022, *19*, 3656

**DOI:** 10.3390/ijerph19116720

**Published:** 2022-05-31

**Authors:** Cézar Akiyoshi Saito, Marco Antonio Bussacos, Leonardo Salvi, Carolina Mensi, Dario Consonni, Fernando Timoteo Fernandes, Felipe Campos, Franciana Cavalcante, Eduardo Algranti

**Affiliations:** 1 Fundacentro, Ministério do Trabalho e Previdência, São Paulo 05409-002, Brazil; cezar.saito@fundacentro.gov.br (C.A.S.); bussacos@fundacentro.gov.br (M.A.B.); fernando.fernandes@fundacentro.gov.br (F.T.F.); 2 Programa de Saúde Ambiental e de Saúde do Trabalhador, Instituto de Saúde Coletiva, Universidade Federal da Bahia, Salvador 40110-040, Brazil; leomaxil@hotmail.com (L.S.); fcampos1205@gmail.com (F.C.); francicavalcante@hotmail.com (F.C.); 3 Occupational Health Unit, Fondazione IRCCS Ca’ Granda Ospedale Maggiore Policlinico, 20122 Milan, Italy; dario.consonni@unimi.it

## Error in Table 1 Title

In the original publication [1], there was a mistake in the title for Table 1 as published. The title has incorrect wording. The two columns “Brazil” refer to the total number of cases of ARD-T, lung and ovarian cancers in adults of 30 years or more and not to “all others”.

The correct title appears below.

Table 1. Number of deaths (*n*) with records of ARD-T, and lung and ovarian cancer in the 29 municipalities (H-ASB) and for all Brazilian municipalities, 2000–2017.

## Error in Table 2 Number

In the original publication [1], there was a mistake in Table 2 as published. The typical asbestos-related diseases (ARD-T) number for men is 357 instead of 257. The corrected Table 2 appears below.

**Table 2:** Number of deaths (*n*), from pooled and individual ARD-T, and lung and ovarian cancer in the H-ASB municipalities and the standardized rate ratios (SRR) and 95% confidence intervals (CI) compared to the reference group, Brazil, 2000–2017. Standard population: Brazil 2010.

Disease (ICD-10 Code)	Men			Women		
	*n*	SRR	95% CI	*n*	SRR	95% CI
Typical asbestos-related diseases (ARD-T)	357	2.56	2.27–2.88	136	1.19	0.99–1.43
Mesothelioma (C45X)	194	1.70	1.45–1.99	114	1.17	0.95–1.43
Asbestosis (J61)	97	6.35	4.86–8.28	14	1.86	0.96–3.39
Pleural plaques (J92.0)	66	5.06	3.69–6.89	8	0.86	0.35–1.79
Lung cancer (C34)	32,604	1.33	1.31–1.34	20,735	1.19	1.17–1.20
Ovarian cancer (C56)	-	-	-	8446	1.34	1.31–1.37

Abbreviations: SRR, standardized rate ratios; CI, confidence interval.

The authors apologize for any inconvenience caused and state that the scientific conclusions are unaffected. The original publication has also been updated.
