# Supplementary material for: Sex-Specific Mortality from Asbestos-Related Diseases, Lung and Ovarian Cancer in Municipalities with High Asbestos Consumption, Brazil, 2000–2017
Source: Int J Environ Res Public Health. 2022 Mar 19;19(6):3656. doi: 10.3390/ijerph19063656 (PMC8949971; doi:10.3390/ijerph19063656)
Supplement: Supplementary file 1 [file ijerph-19-03656-s001.zip › ijerph-1582372-supplementary.pdf]

Supplementary Table S1. Population (2010) and number of deaths with records of ARD-T, and lung and ovarian cancer in the H-ASB municipalities (and respective Brazilian States), Brazil, 2000-2017.

**Municipality**	**Population 2010**			**ARD-T**			**Lung cancer**			**Ovarian cancer**	
	**Men**	**Women**	**Total**	**Men**	**Women**	**Total**	**Men**	**Women**	**Total**	**Women**	**Total**
Belém (Pará)	308,004	372,234	680,238	6	5	11	1730	1116	2846	444	444
Recife (Pernambuco)	354,451	467,032	821,483	19	8	27	2449	1656	4105	835	835
**Arapiraca** (Alagoas)	43,498	51,926	95,424	1	1	2	103	100	203	39	39
**Jaramataia** (Alagoas)	1134	1131	2265	1	0	1	4	1	5	0	0
**Poções** (Bahia)	9877	11,024	20,901	1	0	1	22	8	30	6	6
Simões Filho (Bahia)	24,974	26,995	51,969	1	0	1	90	42	132	29	29
Contagem (Minas Gerais)	146,195	164,703	310,898	7	3	10	620	386	1006	167	167
Pedro Leopoldo (Minas Gerais)	14,727	16,299	31,026	1	0	1	95	42	137	23	23
Duque de Caxias (Rio de Janeiro)	201,148	233,936	435,084	13	6	19	1173	755	1928	257	257
Nova Iguaçu (Rio de Janeiro)	187,780	220,354	408,134	2	5	7	1132	727	1859	250	250
Rio de Janeiro (Rio de Janeiro)	1,603,494	2,016,807	3,620,301	75	52	127	14,963	9253	24,216	3862	3862
Araras (São Paulo)	31,399	33,733	65,132	7	1	8	274	174	448	43	43
Capivari (São Paulo)	12,146	12,637	24,783	2	0	2	79	35	114	17	17
Hortolândia (São Paulo)	46,017	47,475	93,492	1	0	1	206	126	332	41	41
**Itapira** (São Paulo)	19,027	20,163	39,190	1	1	2	133	59	192	29	29
Leme (São Paulo)	22,671	23,558	46,229	22	0	22	161	78	239	17	17
Nova Odessa (São Paulo)	13,449	14,306	27,755	0	0	0	91	32	123	10	10
Osasco (São Paulo)	159,604	185,300	344,904	125	6	131	1022	602	1624	247	247
São Caetano do Sul (São Paulo)	41,719	53,642	95,361	16	6	22	478	249	727	108	108
Colombo (Paraná)	47,850	51,545	99,395	0	0	0	225	174	399	76	76
Curitiba (Paraná)	433,168	518,286	951,454	17	13	30	2864	2033	4897	844	844
Londrina (Paraná)	126,432	147,647	274,079	5	6	11	880	551	1431	242	242
São José dos Pinhais (Paraná)	61,684	65,182	126,866	1	1	2	313	212	525	94	94
Criciúma (Santa Catarina)	47,143	52,118	99,261	7	3	10	480	217	697	56	56

Esteio (Rio Grande do Sul)	20,066	23,444	43,510	6	2	8	266	156	422	29	29
Sapucaia do Sul (Rio Grande do Sul)	32,025	35,707	67,732	4	2	6	418	243	661	39	39
Anápolis (Goiás)	79,291	89,551	168,842	2	2	4	415	321	736	90	90
Goiânia (Goiás)	304,250	359,900	664,150	7	13	20	1877	1368	3245	551	551
**Minaçu** (Goiás)	8147	7759	15,906	7	0	7	41	19	60	1	1
SUBTOTAL	4,401,370	5,324,394	9,725,764	357	136	493	32,604	20,735	53,339	8446	8446
BRAZIL	44,390,356	49,092,675	93,483,031	1686	1035	2721	261,816	157,449	419,265	58,182	58,182

Abbreviations: ARD-T, typical asbestos-related diseases. H-ASB, high asbestos-consumption municipalities. Municipality in bold, municipality with asbestos mining. Municipality in italic, municipality with asbestos cement plant.

Supplementary Table S2. Crude and age-standardized rates (ASRs, per million) in the 29 municipalities (H-ASB) and in Brazil (without H-ASB) by year of death for men 30 years and over in Brazil, 2000-2017.

			ARD-T						Lung Cancer					
	Population		H-ASB			Brazil			H-ASB			Brazil		
**Year**	**H-ASB**	**Brazil**	**n**	**Crude**	**ASR**	**n**	**Crude**	**ASR**	**n**	**Crude**	**ASR**	**n**	**Crude**	**ASR**
2000	3,575,834	31,155,641	15	4.2	4.9	29	0.9	1.0	1717	480.2	577.3	9178	294.6	338.1
2001	3,616,464	32,033,948	17	4.7	4.9	36	1.1	1.3	1641	453.8	546.8	9605	299.8	341.5
2002	3,693,324	32,877,524	15	4.1	4.9	74	2.3	2.6	1753	474.6	556.3	9848	299.5	339.1
2003	3,771,047	33,729,987	29	7.7	8.8	60	1.8	1.9	1650	437.5	508.1	10,345	306.7	344.6
2004	3,851,339	34,607,635	18	4.7	5.5	61	1.8	1.9	1832	475.7	544.8	11,023	318.5	355.7
2005	3,935,584	35,519,606	28	7.1	7.8	56	1.6	1.7	1743	442.9	504.4	11,534	324.7	360.8
2006	4,022,902	36,465,217	18	4.5	5.1	78	2.1	2.3	1779	442.2	498.8	11,727	321.6	355.1
2007	4,113,588	37,443,764	15	3.6	4.4	59	1.6	1.7	1785	433.9	484.2	12,518	334.3	366.5
2008	4,207,414	38,461,079	16	3.8	4.2	80	2.1	2.2	1809	430.0	477.8	12,661	329.2	357.4
2009	4,303,589	39,512,511	19	4.4	4.9	76	1.9	2.0	1784	414.5	452.2	13,027	329.7	354.2
2010	4,401,370	40,585,630	16	3.6	4.1	79	1.9	2.0	1881	427.4	463.4	13,381	329.7	350.7
2011	4,492,199	41,558,921	18	4.0	4.4	106	2.6	2.7	1893	421.4	451.9	13,414	322.8	341.2
2012	4,584,259	42,550,739	31	6.8	7.5	77	1.8	1.9	1893	412.9	437.5	14,040	330.0	346.1
2013	4,676,394	43,553,800	25	5.3	5.6	96	2.2	2.3	1905	407.4	428.4	14,720	338.0	351.5
2014	4,768,130	44,557,719	26	5.5	5.9	98	2.2	2.2	1861	390.3	405.4	15,081	338.5	348.4
2015	4,858,699	45,555,647	12	2.5	2.7	91	2.0	2.0	1843	379.3	387.5	15,400	338.0	344.5
2016	4,941,550	46,553,054	23	4.7	4.7	78	1.7	1.7	1929	390.4	395.5	15,731	337.9	340.7
2017	5,026,091	47,540,976	16	3.2	3.2	95	2.0	2.0	1906	379.2	376.4	15,979	336.1	334.3

Abbreviations: ARD-T, typical asbestos-related diseases. H-ASB, high asbestos-consumption municipalities. ASR, age-standardized rates.

Supplementary Table S3. Crude and age-standardized rates (ASRs, per million) in the 29 municipalities (H-ASB) and in Brazil (without H-ASB) by year of death for women 30 years and over in Brazil, 2000-2017.

			ARD-T						Lung Cancer						Ovary Cancer					
	Population		H-ASB			Brazil			H-ASB			Brazil			H-ASB			Brazil		
**Year**	**H-ASB**	**Brazil**	**n**	**Crude**	**ASR**	**n**	**Crude**	**ASR**	**n**	**Crude**	**ASR**	**n**	**Crude**	**ASR**	**n**	**Crude**	**ASR**	**n**	**Crude**	**ASR**
2000	4,309,361	33,676,111	9	2.1	2.1	26	0.8	0.8	808	187.5	192.2	4161	123.6	131.6	355	82.4	84.5	1776	52.7	55.5
2001	4,365,823	34,700,683	6	1.4	1.3	26	0.7	0.8	809	185.3	186.8	4313	124.3	131.4	382	87.5	87.1	1856	53.5	56.2
2002	4,464,348	35,681,110	7	1.6	1.5	51	1.4	1.5	807	180.8	179.9	4549	127.5	132.6	369	82.7	82.6	2016	56.5	58.6
2003	4,562,969	36,664,148	8	1.7	1.8	40	1.1	1.1	917	201.0	197.2	5028	137.1	141.2	407	89.2	87.7	2057	56.1	57.5
2004	4,663,356	37,668,840	2	0.4	0.4	33	0.9	0.9	937	200.9	194.3	5463	145.0	147.7	411	88.1	86.1	2232	59.3	60.3
2005	4,767,109	38,704,121	8	1.7	1.6	51	1.3	1.3	1000	209.8	200.8	5776	149.2	150.7	418	87.7	84.6	2285	59.0	59.7
2006	4,870,801	39,772,345	7	1.4	1.4	36	0.9	0.9	971	199.4	190.3	6409	161.1	161.5	423	86.8	83.1	2409	60.6	60.8
2007	4,977,484	40,870,736	7	1.4	1.4	49	1.2	1.2	1065	214.0	201.9	6947	170.0	169.0	455	91.4	86.7	2497	61.1	60.9
2008	5,089,502	42,020,442	6	1.2	1.1	57	1.4	1.3	1126	221.2	206.4	7196	171.2	168.6	476	93.5	88.2	2730	65.0	64.2
2009	5,206,169	43,216,055	5	1	0.8	53	1.2	1.2	1174	225.5	206.9	7572	175.2	170.5	511	98.2	91.4	2877	66.6	65.2
2010	5,324,394	44,428,534	13	2.4	2.2	63	1.4	1.4	1223	229.7	208.0	8061	181.4	174.4	498	93.5	86.3	2929	65.9	64.1
2011	5,434,990	45,543,722	6	1.1	1.0	59	1.3	1.2	1171	215.5	194.0	8655	190.0	181.3	483	88.9	81.1	3036	66.7	64.2
2012	5,546,492	46,675,691	12	2.2	2.0	62	1.3	1.3	1312	236.5	209.3	9092	194.8	184.1	519	93.6	85.2	3119	66.8	64.0
2013	5,657,852	47,815,594	9	1.6	1.4	62	1.3	1.2	1373	242.7	213.2	9549	199.7	187.0	533	94.2	84.9	3277	68.5	65.2
2014	5,768,166	48,951,587	8	1.4	1.2	64	1.3	1.2	1454	252.1	216.7	10,202	208.4	193.2	540	93.6	83.6	3340	68.2	64.5
2015	5,876,674	50,075,636	4	0.7	0.6	48	1.0	0.9	1482	252.2	215.9	10,821	216.1	197.6	530	90.2	81.0	3581	71.5	67.2
2016	5,975,763	51,194,135	14	2.3	2.0	61	1.2	1.1	1543	258.2	216.7	11,171	218.2	197.3	590	98.7	86.8	3777	73.8	68.5
2017	6,076,240	52,295,269	5	0.8	0.7	58	1.1	1.0	1563	257.2	212.0	11,749	224.7	200.4	546	89.9	79.3	3942	75.4	69.3

Abbreviations: ARD-T, typical asbestos-related diseases. H-ASB, high asbestos-consumption municipalities. ASR, age-standardized rates.
